# Medicine

**Published:** 1891-09

**Authors:** Wm. C. Krauss


### MEDICINE.

BY

WM. C. KRAUSS, M. D.

In the *Bulletin Général de Thérapeutique* for April 18, 1891, Dr. August Voisin, of Paris, gives his experience with chlorhydrate of morphia in mental and nervous disorders.

*Indications.*—The indications for the treatment of mental alienation apply, above all, to those forms of simple disease, or those freed from congestive complications. It is very useful in hallucinations, in the depressive state, in all forms of alcoholism, suicidal or homicidal motives, etc. In hysterical mania, the author has had excellent results, also in puerperal mania.

It is contra-indicated in all inflammatory forms of mental disease, such as following epilepsy and certain forms of general paresis. It may even be detrimental in cases where the alienation is consecutive to atheromatous changes in the arterial system. The dose is six centigrammes, and more, daily.

---

### NEW REMEDIES AGAINST TUBERCULOSIS.

Since the publication of Koch's treatment of tuberculosis, scores of experimenters have put in claims, believing that the real specific has at last been found in his particular remedy. Not to mention the Gibbes-Shurly and Liebrich treatments, we find in recent literature such remedies as goat blood injections, hypodermic injection of oil of creosote, oil of guaiacol, thymolo-acetate of mercury, along with the internal administration of iodide of potassium, inhalation of chloroform and ether, etc., etc. The life of the tubercle bacillus can only be destroyed by carefully studying its biological relations, and we believe, therefore, that the Koch plan is one which will eventually succeed.

---

Killsbren, in an article on Chloralamid, in the *Memphis Medical Monthly*, makes a comparison between chloralamid and chloral on one hand, and sulfonal on the other. His results are as follows:

Compared with chloral:

1. It is more agreeable to the taste, and consequently easier administered.
2. Rarely causes digestive disorders.
3. Does not depress the heart or circulation.
4. Seldom produces cerebral disturbances.

As compared with sulfonal:

1. Much more prompt in its action.
2. Being more soluble, it can be administered easier.
3. Sleep nearly always passes away by morning.
4. It is only about one-half as expensive.

---

### THE PATHOLOGICAL ANATOMY OF TIC DOULOUREUX.

In *The Journal of Nervous and Mental Disease*, Vol. XVI., p. 54, Dr. C. L. Dana, of New York, asserts that trigeminus neuralgias are not due to neuritic or degenerative processes, but to an arterio-sclerosis, which limits the supply of blood to the nerve. In four cases in which he excised a portion of the trigeminus, no organic process in the nerve could be demonstrated. In three of these, however, sclerotic patches were present in the vessels. Therapeutically he recommends agents which act on the vaso-motor system, such as aconitin, nitro-glycerin, etc.

